# Advances in the Development of Biomaterials for Endotoxin Adsorption in Sepsis

**DOI:** 10.3389/fbioe.2021.699418

**Published:** 2021-07-30

**Authors:** Qinbo Yang, Yupei Li, Pazilaiti Tuohuti, Zheng Qin, Zhuyun Zhang, Weifeng Zhao, Baihai Su

**Affiliations:** ^1^Department of Nephrology, National Clinical Research Center for Geriatrics, West China Hospital, Sichuan University, Chengdu, China; ^2^Disaster Medicine Center, Institute for Disaster Management and Reconstruction, Sichuan University, Chengdu, China; ^3^West China School of Medicine, Sichuan University, Chengdu, China; ^4^State Key Laboratory of Polymer Materials Engineering, College of Polymer Science and Engineering, Sichuan University, Chengdu, China; ^5^Med-X Center for Materials, Sichuan University, Chengdu, China; ^6^The First People's Hospital of Shuangliu District, Chengdu, China

**Keywords:** endotoxin, lipopolysaccharides, sepsis, adsorbent, hemoperfusion

## Abstract

Sepsis, a life-threatening and intractable disease without any specific treatment, is activated by endotoxin. Some attempts at removing endotoxin to treat sepsis from the blood circulation using different hemoperfusion cartridges have been proposed recently, but they have failed to reduce the mortality of severe septic patients. This review summarizes the latest advances in the development of endotoxin adsorbents. In particular, we highlight two critical parameters for endotoxin adsorbents when they are applied in blood purification: the dissociation constant and the maximum adsorption capacity. We also discuss potential challenges and research directions for the future development of endotoxin adsorbents.

## Graphical Abstract

Overview schematic of endotoxin adsorbents. 
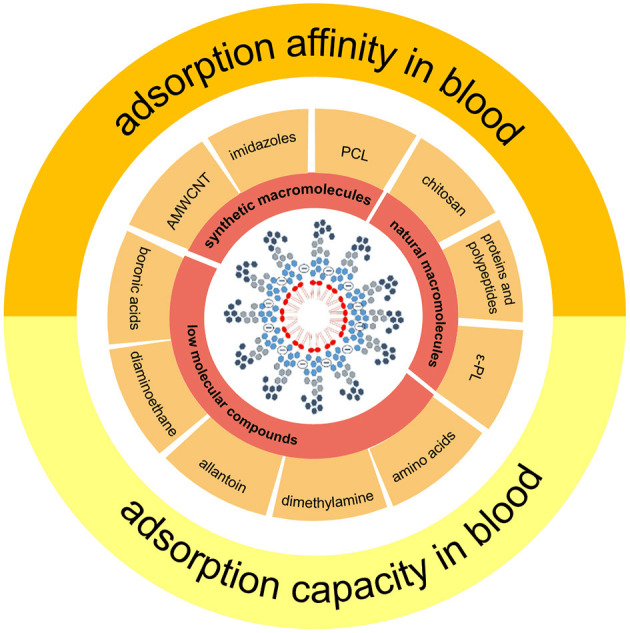

## Introduction

Sepsis is caused by an imbalanced host response to infection and can rapidly lead to life-threatening organ dysfunction (Singer et al., 2016). It is a significant healthcare concern with a substantial global burden associated with high mortality and increased healthcare costs (Torio and Andrews, 2013; Fleischmann et al., 2016). Even though remarkable advances have been made in the early diagnosis and implementation of sepsis bundles recommended by the Surviving Sepsis Campaign (Rhodes et al., 2017), the mortality of patients with severe sepsis and septic shock remains high 90-day mortality of 35.5% in the intensive care unit (Xie et al., 2020).

Endotoxin (or lipopolysaccharide), a component of the outer wall of Gram-negative bacteria, is one of the most important pathogen-associated molecular patterns (PAMPs) in sepsis (Lelubre and Vincent, 2018). After infection, the invading pathogen significantly activates the host innate immune system, which is mediated by activating pattern recognition receptors by endotoxin and other PAMPs, to induce early local immune response for pathogen elimination (van der Poll et al., 2017). In most cases, the innate immune system is efficient in mounting a protective and balanced response to infections, which results in the elimination of the pathogen through a series of pro-inflammatory reactions such as the release of cytokines and chemokines, the recruitment of phagocytes, and the local activation of the complement and coagulation systems, followed by a return to homeostasis (van der Poll et al., 2017). However, it would be harmful if invasive pathogens continuously caused stimulation resulting in immunological imbalances and converting usually beneficial inflammation into adverse reactions. In this case, excessive inflammation, known as a “cytokine storm,” occurs and manifests as cellular injury, catabolism, and multiple organ dysfunction, while sepsis-associated immunosuppression accelerates apoptosis of immunizing cells to make the host vulnerable to pathogens (van der Poll et al., 2017). Although conventional treatments such as antibiotic therapy and organ function support can delay the exacerbation of this disease to some extent, they undoubtedly overlook the immunopathological nature of sepsis. As a result, the mortality associated with severe sepsis and septic shock demonstrates an increasing trend (Gotts and Matthay, 2016; Rhodes et al., 2017).

High endotoxin levels are associated with multiple organ failure and mortality in sepsis (Klein et al., 2007). Although endotoxin-targeted medicines failed to show improved outcomes for septic patients, substantial evidence suggests that the removal of endotoxin by extracorporeal techniques represents an attractive area for research (Davies and Cohen, 2011; Marshall, 2014; Ankawi et al., 2018). The use of conventional continuous renal replacement therapy modalities such as high volume hemofiltration (Joannes-Boyau et al., 2013; Borthwicka et al., 2017), very high volume hemofiltration (Ankawi et al., 2018), and high cut-off membranes (Chelazzi et al., 2016), cannot effectively remove endotoxin from blood circulation and has yielded inconsistent results in mortality reduction and hemodynamics improvement. Alternatively, hemoperfusion can be performed. This involves the passage of blood through hemofilters where cytokines or endotoxin is adsorbed to membrane surfaces or through sorbent-containing cartridges. During this process, both the dissociation constant between endotoxin-binding ligands and endotoxin molecules and the maximum endotoxin adsorption capacity determine the endotoxin adsorption efficacy of such hemofilters or cartridges. Polymyxin B-immobilized fiber (PMX®) is the most commonly used material in endotoxin adsorption columns. Polymyxin B (PMB) is a cationic antimicrobial peptide from *Bacillus polymyxa* that can bind and neutralize endotoxin (Harm et al., 2019), but early clinical evidence for PMX® showed paradoxical results on clinical endpoints (Iwagami et al., 2014, 2016; Payen et al., 2015). More recently, the EUPHRATES randomized controlled trial with a larger sample size merely demonstrated that the use of PMX® resulted in increased mean arterial pressure for overall septic patients as evidenced by the reduced requirements for vasoactive drugs. Meanwhile, the mortality significantly decreased from 41.9 to 20% when the target population was narrowed to patients with endotoxin activity assay (EAA) measurements between 0.6 and 0.9 (Dellinger et al., 2018; Klein et al., 2018). In contrast, this beneficial effect on mortality cannot be found in patients with an EAA higher than 0.9, suggesting the insufficient capacity of PMX® to control the endotoxin burden in more critically ill patients with sepsis. Notably, some researchers argued that the PMX® cartridge also worked as a carrier, because the reduced endotoxin might be caused by the non-covalently bound PMB molecules that leaked from a PMX® cartridge into the blood (Harm et al., 2014). Another hemoperfusion adsorption column for endotoxin removal in extracorporeal circulation is the Alteco LPS Adsorber® that is filled with polyethylene plates with an endotoxin-adsorbing peptide. Clinical studies with small sample sizes showed that the use of Alteco LPS Adsorber® was associated with increased blood pressure and reduced vasopressor requirements, resulting in the effective elimination of endotoxin from the blood of patients with septic shock (Blomquist et al., 2009; Ala-Kokko et al., 2011; Adamik et al., 2015).

Beyond endotoxin, various pro-inflammatory and anti-inflammatory cytokines also play essential roles in sepsis, as they can activate or impair sepsis-related inflammatory reactions (Gotts and Matthay, 2016; Malard et al., 2018; Honore et al., 2019). The levels of cytokines in sepsis have been found to correlate with the endotoxin concentration (Ronco and Klein, 2014). Thus, therapeutic strategies aiming to eliminate inflammatory cytokines emerge as an appealing method. CytoSorb®, which is a hemoadsorption device containing porous polystyrene-divinylbenzene copolymer beads capable of removing cytokines by size exclusion and surface adsorption, significantly decreased a broad spectrum of cytokines by more than 50% (Gruda et al., 2018; Malard et al., 2018; Ankawi et al., 2019). A prospective study by Friesecke et al. found that cytokine adsorption with CytoSorb® was accompanied by hemodynamic and metabolic stabilization (Friesecke et al., 2017). However, a more recent randomized controlled trial did not observe an association of CytoSorb® hemoperfusion with mortality in mechanically ventilated patients with severe sepsis or septic shock (Schädler et al., 2017). AN69 is a filtration membrane with high-efficiency adsorption to cytokines (Honore et al., 2013), while the oXiris® filter is modified with the AN69 membrane whose surface is further treated by layers of polyethyleneimine (PEI) and heparin, making itself capable of adsorbing both endotoxin and cytokine molecules (Shum et al., 2013). oXiris® exhibited similar endotoxin adsorption to PMX® and similar cytokines adsorption to CytoSorb®, significantly reducing endotoxin and cytokines and improving organ function for patients with sepsis in several case studies. However, less is known about its effect on mortality (Adamik et al., 2013; Shum et al., 2013; Malard et al., 2018; Broman et al., 2019). Consequently, attempts to treat sepsis by endotoxin adsorption partially ameliorate symptoms of patients, but most importantly, they are not sufficient to prevent progression and long-term outcome of patients with sepsis. To address these drawbacks, a considerable number of studies have been attempted. Herein, we set out to review the latest advances in developing endotoxin adsorbents and discuss the challenges for endotoxin removal from the blood.

## Mechanisms of Endotoxin Adsorption

**Figure 1:**
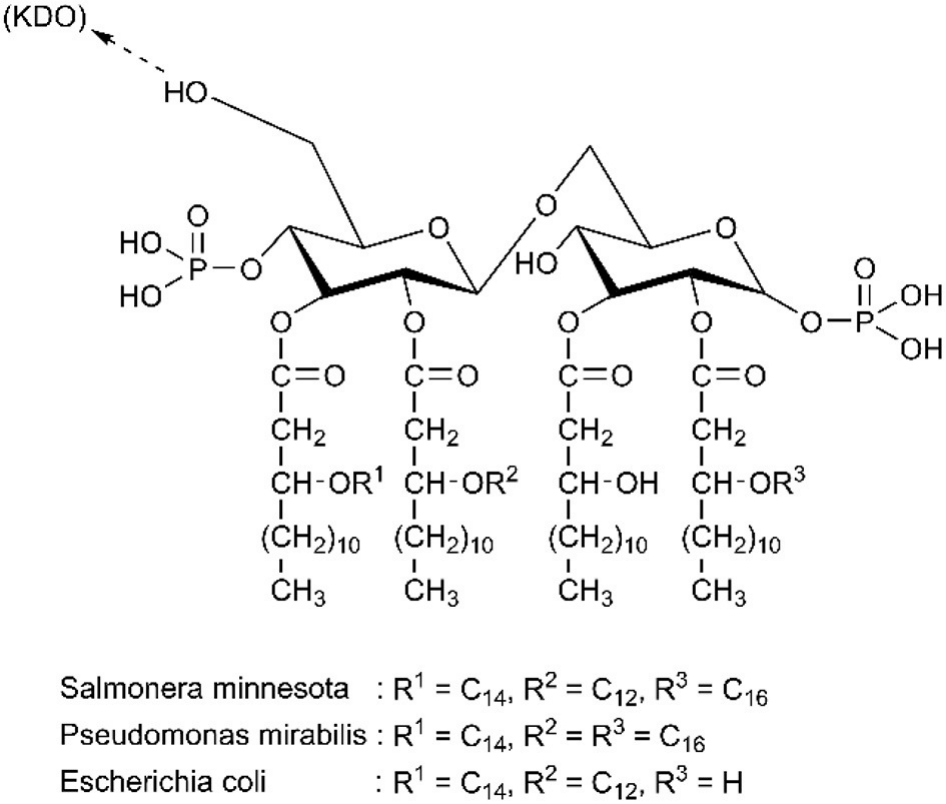
Chemical structure of diverse lipid A. Reproduced with permission of Elsevier (Hirayama and Sakata, 2002).

Understanding the structure of endotoxin in the blood is an important and challenging step, and it can directly improve the design of adsorbents. Endotoxin is composed of a hydrophilic heteropolysaccharide component and a covalently bound lipid moiety. Even though the structure and molecular weight of various endotoxins from different bacterial sources vary significantly, they all contain lipid A, which is considered the smallest unit of endotoxic activity (Magalhaes et al., 2007). Figure 1 shows that lipid A is partially phosphorylated, whose hexosamine groups are highly substituted with long-chain fatty acids. Therefore, endotoxin is an amphiphilic substance containing both anionic and hydrophobic groups (pK_1_ = 1.3, pK_2_ = 8.2) (Hirayama and Sakata, 2002).

**Figure 2:**
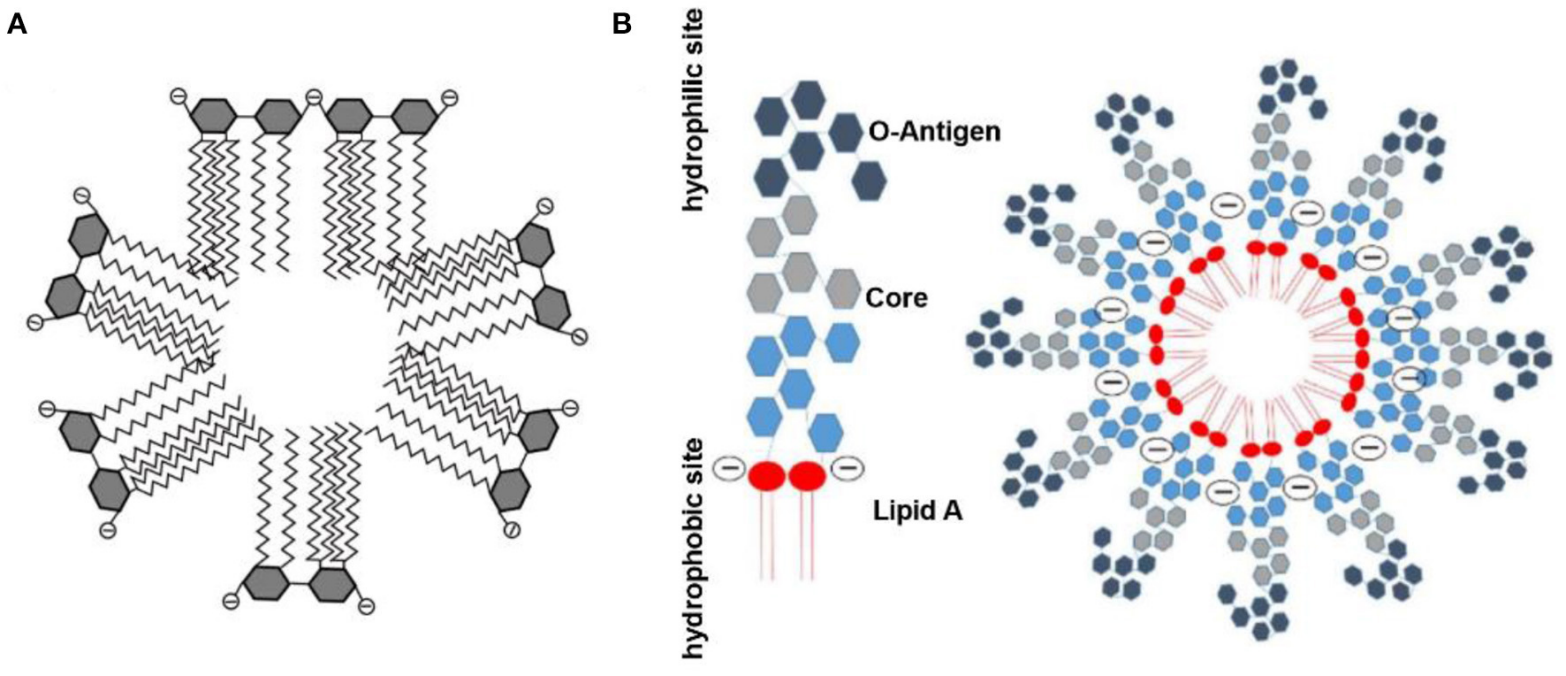
Schematic diagram of endotoxin assembly in solution. **(A)** Reproduced with permission of Elsevier (Gorbet and Sefton, 2005). **(B)** Reproduced with permission of Springer Nature (Harm et al., 2021).

The requirement for inexpensive and efficient endotoxin removal has enhanced development efforts for various biomaterials with endotoxin-binding activity. Among the diverse methods for endotoxin removal, affinity adsorption is regarded as the most effective technique that benefits from selective and robust interaction between endotoxin and the endotoxin-binding ligands immobilized on diverse matrices (Schneier et al., 2020). In aqueous solutions, endotoxin aggregates as a supramolecular assembly because of its amphiphilic nature. London et al. suggested that the phosphate groups of monomeric endotoxin can form the outer layer of this supramolecular assembly (Figure 2A), and that the other parts form the inner one, which allows the endotoxin aggregates to interact with cationic adsorbents (London et al., 2014). In contrast, Harm et al. proposed that lipid A inserted into the aggregate interior, with the hydrophilic sites as head groups (Figure 2B) (Harm et al., 2021). These aggregate structures exhibited high stability even at extreme pH values and temperatures. However, there is no definitive information concerning the aggregate structure and size of endotoxin in human blood (Hirayama and Sakata, 2002). Endotoxin adsorption from some matrices of adsorbents also indicates a hydrophobic bond with endotoxin, which would be compromised by the complex solution components (Todokoro et al., 2002). Since the affinity of PMB to endotoxin has been attributed to the simultaneous effects of hydrophobic and cationic groups in PMB molecules (Thomas et al., 1998), amphiphilic materials have been designed as selective ligands to endotoxin.

It is noteworthy that endotoxin removal in whole blood distinguishes from that in drugs and protein solutions where selective adsorption could be achieved through environmental changes. There are both acidic and hydrophobic proteins in plasma, so adsorbents with excessive hydrophobicity or cation city will inevitably impact the plasma protein contents. As the most potent pyrogen, the administration of very low doses of endotoxin leads to clinical symptoms, and more than 4 ng/ml of endotoxin in the blood is an extreme burden for patients with severe sepsis (Machado et al., 2006; Romaschin et al., 2017; Klein et al., 2018). The biggest challenge for adsorbent use is enabling high endotoxin adsorption selectivity under physiological conditions and cleansing endotoxin from low to negligible levels with no side effects. However, because of the complexity in adsorption mechanisms, the quantitative determination of affinity to endotoxin will help to compare and screen adsorbents (Basauri et al., 2020). Kinetic studies employing either surface plasmon resonance, microcalorimetry, or fluorescence resonance energy transfer have, thus, been performed to analyze dissociation constants (K_d_) between endotoxin and different molecules (Viriyakosol et al., 2001; Shin et al., 2007). K_d_ decreases with enhanced affinity to endotoxin, and the exceptionally high affinity is expected to adsorb endotoxin at low concentrations (Hirayama and Sakata, 2002). In most studies, adsorbents for endotoxin followed the Langmuir adsorption isotherm, demonstrating monolayer formation of endotoxin onto the adsorbent surface and in which K_d_ and maximum adsorption capacity (Q_m_) were indirectly calculated. Q_m_ denotes the adsorption capacity when all functional groups are saturated. Both Q_m_ and K_d_ are considered parameters to develop new endotoxin adsorption techniques (Basauri et al., 2020). In the following section, we classify adsorbents by molecules of ligands and into the following groups: natural macromolecules, synthetic macromolecules, and low molecular compounds.

## Adsorbents for Endotoxin Removal

### Natural Macromolecules

#### Chitosan

**Figure 3:**
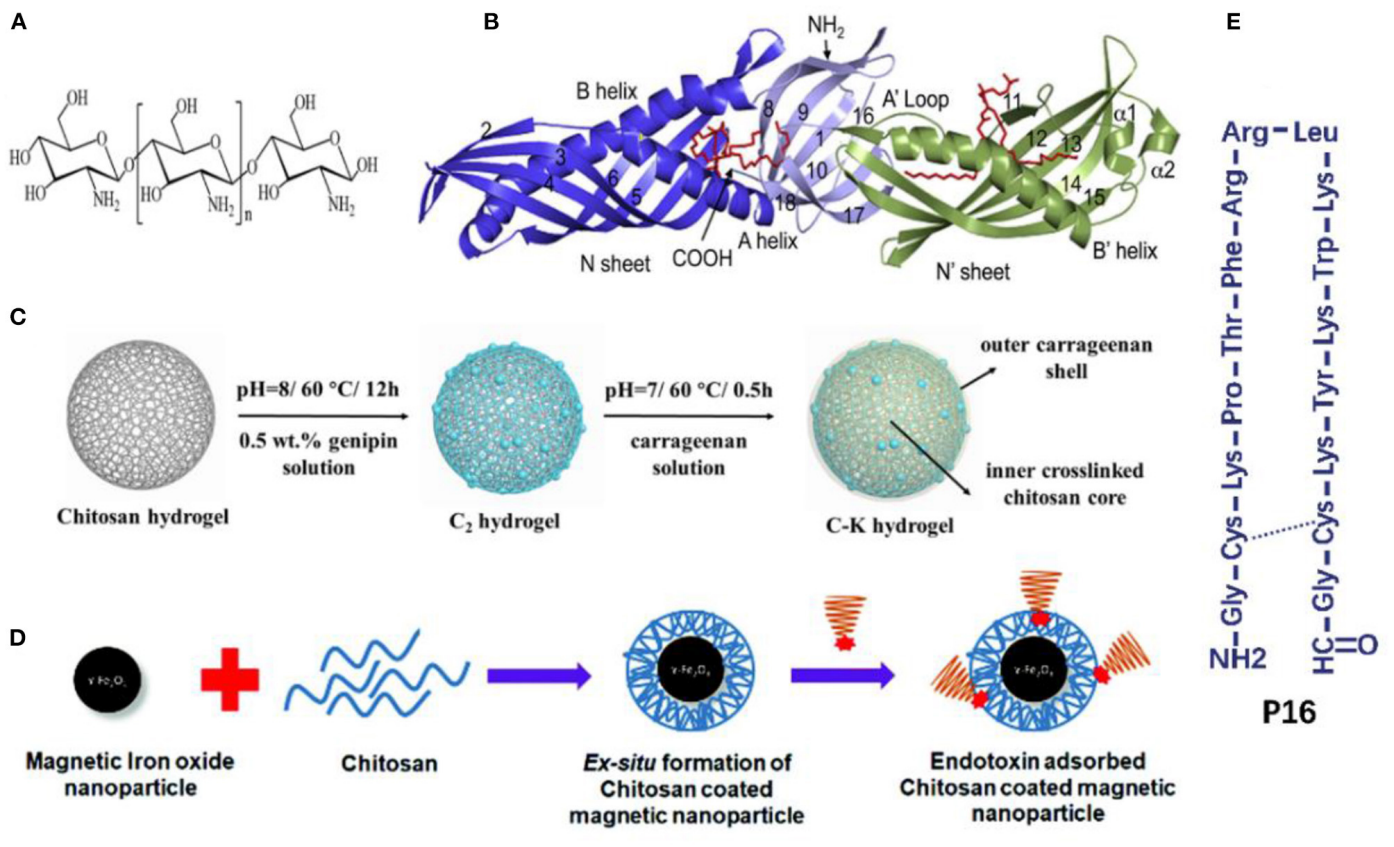
**(A)** Chemical structure of chitosan. Reproduced with permission of Royal Society of Chemistry (Konwar et al., 2019). **(B)** Crystal structure of the LBP, the red indicates phospholipid molecules. Reproduced with permission of Elsevier (Eckert et al., 2013). **(C)** Schematic illustration of the fabrication process of carrageenan-immobilized genipin-crosslinked chitosan hydrogels. Reproduced with permission of Elsevier (Li et al., 2020). **(D)** Chitosan-coated magnetic iron oxide nanoparticles. Reproduced with permission of Royal Society of Chemistry (Konwar et al., 2019). **(E)** Schematic illustration of P16. Reproduced with permission of Elsevier (Tang et al., 2020).

Natural macromolecules are polymers that come from natural sources or can be synthesized from naturally sourced materials (Gusain et al., 2020). Chitosan is the second most abundant natural polymer (Fortunato et al., 2019) that enriches amino groups capable of binding with negatively charged endotoxin by electrostatic interactions (Figure 3A). Some studies also revealed that the formation of the chitosan-endotoxin complex depends on hydrogen bonds (Konwar et al., 2019). De Freitas et al. discovered that the chitosan membrane significantly reduced the level of endotoxin by 91–97% in buffer and protein solutions with high endotoxin concentrations of 700–900 EU/ml, whereas the protein recoveries varied from 65 to 99% resulting from protein trapping in membrane pores (de Freitas et al., 2004; Machado et al., 2006). Sun et al. immobilized chitosan on a poly (vinylidene fluoride) (PVDF) hollow fiber membrane (Sun et al., 2005). Q_m_ and K_d_ of the chitosan-PVDF membrane, calculated with Langmuir adsorption isotherms, were 21.4 EU/mg and 0.5 EU/ml. Dynamic adsorption experiments in the membrane cartridge were performed with the single-pass filtration mode. After filtration at 1 ml/min for 20 min, endotoxin with an initial concentration of 5 EU/ml was removed by nearly 85% (estimated from the figures in the published study). Meanwhile, competing interactions did occur in protein solutions. Acidic proteins such as bovine serum albumin (BSA) combined with binding sites at the adsorbent, while basic proteins such as lysozyme competed for endotoxin molecules and affected removal efficiency. In the previous study (Li et al., 2020), genipin-crosslinked chitosan hydrogels were synthesized for endotoxin removal from the blood. The doping of carrageenan onto the surface of the chitosan hydrogels significantly prolonged the clotting times of plasma, making it possible to use these chitosan-based adsorbents in anticoagulant-free hemoperfusion sessions (Figure 3C). The obtained adsorbent exhibited excellent endotoxin adsorption capacity (202.8 EU/g in PBS solutions and 114 EU/g in simulative septic blood). Additionally, the bacterial burden in blood was also suppressed after a short-time contact between the blood and the obtained endotoxin adsorbent, which had the antimicrobial properties of chitosan. It is noteworthy that although chitosan is found to inhabit lipopolysaccharide (LPS)-induced inflammation, it can also be proinflammatory by triggering the activation of inflammasomes (Gudmundsdottir et al., 2015; Shi et al., 2019).

#### Proteins and Polypeptides

Natural endotoxin receptors in human beings and other creatures are peptide-based structures, part of them engaging in the recognition and transport of endotoxin (Basauri et al., 2020). Some proteins and polypeptides exhibit high affinities to endotoxin with good safety profiles *in vivo*, making them highly desirable for developing endotoxin adsorbents. This section discusses the recent advances in endotoxin adsorbents that are prepared by proteins, such as LBP, lysozyme, and polypeptides, such as FcMBL and PMB.

Human lipopolysaccharide-binding protein (LBP), produced by the liver, is the most extensively explored endotoxin-bond protein, which presents the highest affinity to endotoxin (Basauri et al., 2020). LBP contains two domains (N-terminal and C-terminal): each contains a single hydrophobic pocket; and the N-terminal domain also contains positively charged residues at the tip (Figure 3B) (Theofan et al., 1994; Eckert et al., 2013). The association constant (K_a_, the reverse of K_d_) between LBP and endotoxin ranges from 1.4 × 10^8^ M^−1^ to 2.88 × 10^8^ M^−1^ in kinetic studies, which is higher than that between PMB and endotoxin (Basauri et al., 2020). Accordingly, Li et al. coated LBP onto polyhydroxyalkanoate particles to reduce endotoxin from 50 to 0.05 EU/ml in protein solutions at pH 8, indicating high affinity was maintained even at low levels of endotoxin (Li et al., 2011a). The adsorption capacity, independent of ionic strengths, decreased by 10–40% when pH fluctuated from 4 to 7. Moreover, the adsorption process was conducted at 4°C overnight, which was not feasible for clinical hemoperfusion practice.

Lysozyme is a bacteriolytic enzyme mainly against Gram-positive bacteria, and is suggested to have the ability to bind with endotoxin (Daniel et al., 2015). Levashov et al. reported that lysozyme-immobilized agarose beads were effective adsorbents for endotoxin removal from a phosphate buffer solution (Levashov et al., 2019a), and 100 μl of adsorbent slurry in 1 ml buffer solution reduced endotoxin from 50 to 5.9 ng/ml. The adsorption results were in line with the Henry isotherm equation by which 200 ml of the adsorbent slurry was estimated to remove more than 90% of endotoxin in 1 L of solution. Moreover, it is also noteworthy that the adsorbent was preserved at a cold temperature to maintain its activity. The presence of endotoxin-bound proteins on adsorbents might ensure excellent affinity to endotoxin if their activity can be retained.

Inspired by the spleen structure, Kang et al. designed a mannose-binding lectin (MBL)-based device *via* a combined micromagnetic-microfluidic technique called the biospleen (Kang et al., 2014). MBL is a blood opsonin that captures pathogens such as microorganisms and endotoxin to the spleen for further phagotrophy. Natural MBL was genetically engineered as FcMBL by deleting the domains that might activate macrophages and fusing the residual domains with IgG1 Fc. Subsequently, FcMBL was coated onto magnetic nanobeads to constitute magnetic opsonins. Upon introducing magnetic opsonin and heparin into whole blood that flowed from septic rats to the extracorporeal circuit, pathogens and endotoxin were fully adsorbed within 5 h. A magnetic separator unit could finally recycle more than 99% of magnetic opsonins before the cleansed blood returned to the rats. Notably, histological analysis revealed that reducing blood pathogen levels using magnetic opsonins also resulted in a significant decrease in both pathogen load and the level of inflammatory cell infiltrate and interstitial edema in the lung, spleen, and kidney. Extended short-term survival of rats with acute endotoxemic shock induced by a lethal dose of endotoxin was observed. However, the impact of tiny amounts of magnetic nanobeads that enter the blood circulation remains unconfirmed. Besides, the clinical application of this unique device was impeded because of its complexity in synthesis and high cost. The same group later prepared an FcMBL-based polysulfone hollow fiber by EDC (1-ethyl-3-(3-dimethylaminopropyl) carbodiimide) chemistry to address these issues (Didar et al., 2015). Bacteria and endotoxin were removed effectively and rapidly by more than 90% even if the initial concentrations of endotoxin were different, both *in vivo* and *in vitro*.

Although the benefit of PMX® is sometimes limited as outlined above, PMB is still an attractive ligand for endotoxin adsorption, which has been loaded onto various matrix materials. For instance, PMB was successfully conjugated with a nanofiber sponge made of polyacrylonitrile nanofiber and SiO_2_ nanofiber (Huang et al., 2019), in which Q_m_ was calculated as 17,889 EU/g from the Langmuir equation, and the endotoxin removal rate reached 90% in human plasma at a low concentration. Gellan-polylysine complex fiber was also used as the matrix for PMB, which exhibited a Q_m_ of 2,784 EU/g (Peng et al., 2020). Besides, Lambadi et al. prepared PMB-capped silver nanoparticles as potential endotoxin adsorbents, which not only removed 97% of endotoxin in aqueous solution remarkably but also enhanced the antimicrobial activity of the silver nanoparticles against multiple drug-resistant clinical strains. This study established the knowledge about the synergistic mode of action of silver nanoparticles with PMB (Lambadi et al., 2015). By loading PMB on polysulfone microtube array membranes, the endotoxin removal rate of the PMB-immobilized adsorbent in human plasma was significantly higher than that of PMX® (89.33 vs. 65.52%) (Chew et al., 2020). It follows that PMB is still a promising substrate for endotoxin removal, and that matrices can play a significant role in the capacity of adsorbents. However, the clinical use of PMB is limited by its nephrotoxicity and neurotoxicity, which remain a significant safety concern for septic patients (Bhattacharjya and Straus, 2020).

P16, an amphiphilic, positively charged domain from the *Limulus* anti-LPS factor, is a cyclic peptide with high binding affinity and specificity toward endotoxin (Mora et al., 2006). Figure 3E shows that P16 contains an amidogen and four lysine monolayers. Tang et al. conjugated P16 with amine-functionalized mesoporous silica nanospheres (MSP) to clear endotoxin successfully (Tang et al., 2020). After a simple multi-cycle removal (~2 or 3 cycles) with 1 mg/ml of MSP-P16 nanospheres each time, endotoxin was wholly removed (decrease from around 12,500 to <0.5 EU/ml).

#### Other Cationic Polymers

It is reported that the endotoxin removal ability of polyamino acid-based adsorbents was associated with the number of amino groups in the polyamino acid molecules (Todokoro et al., 2002). Therefore, ε-Poly-L-lysine (ε-PL) encompassing 25–30 L-lysine residues with positively charged amine groups was covalently immobilized onto chloromethyloxirane-activated cellulose particles and used for endotoxin adsorption from the protein solution (Todokoro et al., 2002; Yu et al., 2010). ε-PL showed a greater affinity to endotoxin (K_d_ = 1.1 × 10^−11^ M), which is lower than that of both PMB and chitosan. Selectivity of the ε-PL-adsorbent was more dependent on the apparent pK_a_ and amino-group content of the ligand, but recovery of BSA increased with the narrowed pore size of the matrix (Sakata et al., 2001). When pore size was below the molecular weight of BSA, the entry of BSA into the pores of the adsorbent could be impeded with endotoxin being strongly adsorbed on the adsorbent surface, because endotoxin has a lower pK_a_ than BSA. Thus, the adjustments to apparent pK_a_ and pore size allow a better selectivity of endotoxin. Even though adsorption capacity was dependent on environmental ionic strength and pH values, the optimum condition of ε-PL-adsorbent was similar to the blood environment. In addition, ε-PL has become available as a preservative, which shows low toxicity and significant antimicrobial spectrum through interfering with membrane integrity, oxidative stress, and gene expression in bacteria (Ye et al., 2013). These properties make ε-PL an ideal candidate for endotoxin affinitive ligand in the blood.

Proanthocyanidins (PACs), a group of naturally occurring plant metabolic polymers composed of monomeric flavonoid subunits, have been reported to possess a variety of bioactivities such as antioxidant, antibacterial, and anti-atherosclerotic activities (Johnson et al., 2008; Ou and Gu, 2014). Delehanty et al. found that PACs could bind to endotoxin and inhibit the interaction between endotoxin and its receptors on mammalian cells to alleviate the endotoxin-induced activation of the NF-κB pathway, a crucial contributor to abnormal inflammation amplification (Delehanty et al., 2007; van der Poll et al., 2017). By comparison, the PACs-immobilized sepharose beads showed a binding affinity for endotoxin similar to that of PMB (Johnson et al., 2008).

Table 1 shows that the applications of natural macromolecules for biomaterials are fascinating because of their advantages. Chitosan, lysozyme, and ε-PL can remove endotoxin efficiently and act on bacteria or cytokines simultaneously, suggesting that they can be used for multi-target therapy for sepsis. Proteins and polypeptides revealed their full ability as ligands to remove endotoxin at low concentrations. Nevertheless, the safety and synthesis cost of endotoxin adsorbents should be considered to affect the translation of such adsorbents to clinical practice.

**Table 1:** Advantages and disadvantages of various adsorbents.

**Ligand**	**Matrix**	**Advantages**	**Disadvantages**
Chitosan	Chitosan	Ease, cost-effective, versatility	Undefined immunogenicity
LBP	PHA particle	High affinity, high efficiency	Cryopreservation, applicability problems
Lysozyme	Agarose bead	High efficiency, versatility	Cryopreservation, applicability problems
FcMBL	Magnetic nanobeads	High affinity, high efficiency, versatility	Technical complexity, high cost
Plymyxin B	Nanofiber sponge	High efficiency, high capacity	Technical complexity, nephrotoxicity and neurotoxicity, high cost
Plymyxin B	Silver nanoparticles	Versatility, high efficiency	Nephrotoxicity and neurotoxicity, unknown biocompatibility, high cost
ε-polylysine	Cellulose particles	Ease, cost-effective, great selectivity, versatility	Unknown biocompatibility, absent blood adsorption test
Poly-ε-caprolactone nanoparticles	Cellulose membrane	Low chemicals required, high efficiency, greater selectivity	Unknown biocompatibility, absent blood adsorption test
Poly (1-vinyl imidazole)	Silica gel particles	High affinity, high efficiency	Unknown biocompatibility, absent blood adsorption test
MDMIBr	Magnetic particles	Versatility, good biocompatibility	Chemicals needed, low capacity
AMWCNT	Polyvinyl alcohol microspheres	High efficiency, great selectivity	Unknown biocompatibility, absent blood adsorption test
L-serine	Polyethersulfone membrane	High affinity, great selectivity, versatility, unique structure with endotoxin	Unknown biocompatibility
Phenylalanine	Polystyrene microspheres	Good biocompatibility	Low capacity
Dimethylamine	Graphene oxide	high efficiency, unique structure with endotoxin, diverse adsorption mechanisms	Unknown biocompatibility, absent blood adsorption test, chemicals needed, tedious process
Allantoin	–	High affinity, low chemicals required	Absent matrix, protein loss
Aminoethane	Cellulose nanofiber	High affinity, greater selectivity, high efficiency	Unknown biocompatibility, absent blood adsorption test
Boronic acids	Magnetic microspheres	Unique adsorption mechanism, high affinity	Unknown biocompatibility, absent blood adsorption test, low capacity

*LBP, lipopolysaccharide-binding protein; PHA, polyhydroxyalkanoate; FcMBL, mannose-binding lectin fused with IgG1 Fc; MDMIBr, 1-(12-mercaptododecyl)-3-methylimidazolium bromide; AMWCNT, amino multi-walled carbon nanotube*.

### Synthetic Macromolecules

#### Poly-ε-Caprolactone

**Figure 4:**
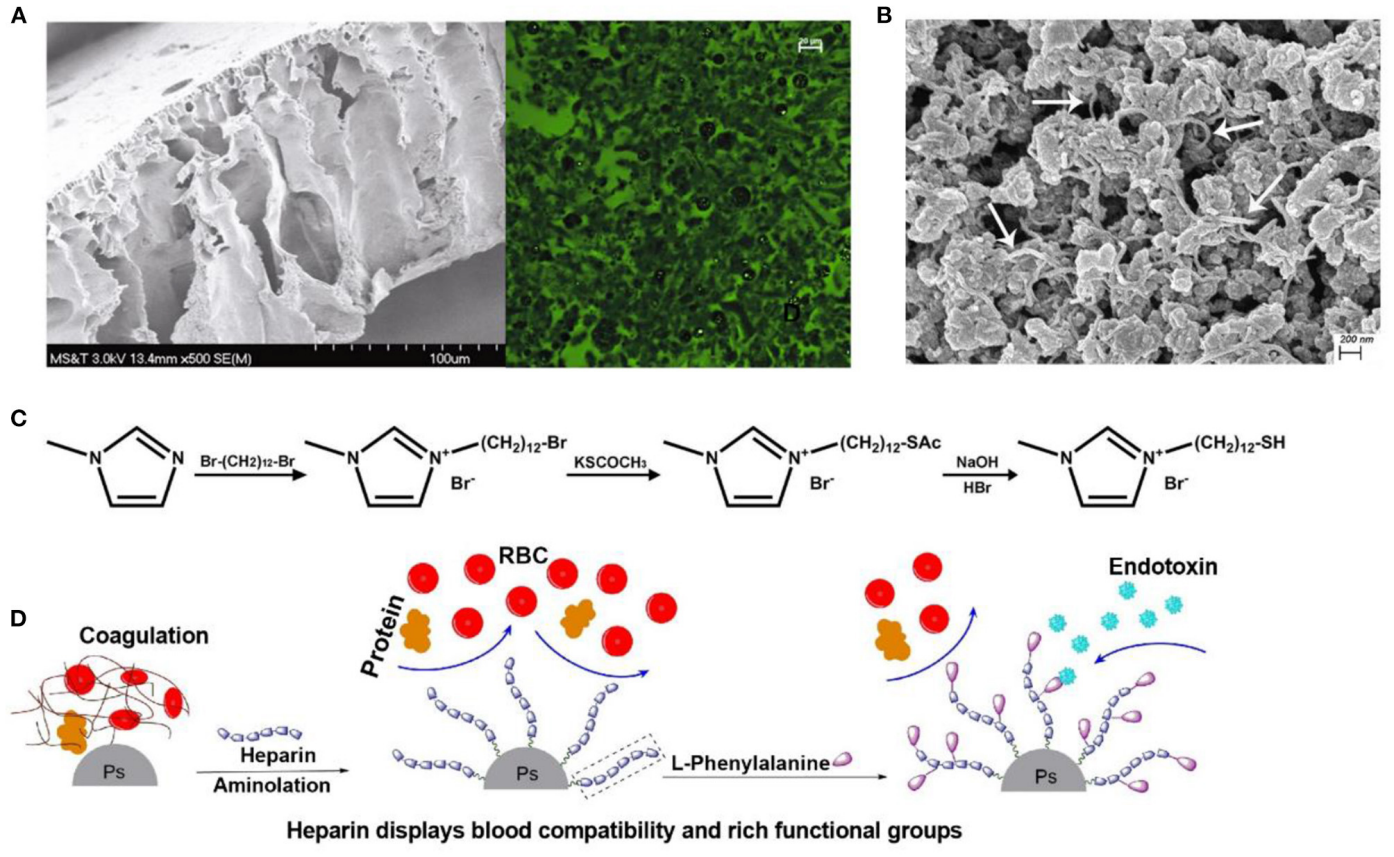
**(A)** Cellulose acetate membrane with PCL NPs in scanning electron microscope and fluorescence microscopic images. PCL NPs were encapsulated with fluorescein dye. Reproduced with permission of Springer Nature (Razdan et al., 2019). **(B)** Scanning electron microscope of PVA-AMWCNT microspheres. The white arrows indicate AMWCNT. Reproduced with permission of Informa Healthcare (Zong et al., 2017). **(C)** Synthesis of MDMIBr. Reproduced with permission of Elsevier (Shi et al., 2020). **(D)** Schematic illustration of heparin and Phe on the surface of adsorbent. Reproduced with permission of Elsevier (Dang et al., 2019).

Poly-ε-caprolactone is an aliphatic polyester whose affinity to endotoxin arises from strong hydrophobic and *van der Waals* interaction (Donnell et al., 2016; Razdan et al., 2019). Donnell et al. synthesized poly-ε-caprolactone (PCL) nanoparticles (PCL-NPs) with an average diameter of 0.4 μm by a simple one-step phase separation process, and 50 μg/ml of PCL-NPs removed 78.8% of endotoxin when the initial concentration was 5 × 10^5^ EU/ml. Razdan et al. further mixed the obtained PCL-NPs into cellulose solution to produce a PCL-NPs-based cellulose membrane for the first time, whose adsorption capacity was ~2-fold higher than that of PCL-NPs alone (Figure 4A) (Razdan et al., 2019). These findings provide the probability that PCL-NPs and NP-based portable filters might be novel, feasible tools for hemoperfusion.

#### Imidazoles

Some endotoxin-bound materials benefit from the hydrogen bond between the nitrogen atom at imidazole rings and endotoxin (Zhang et al., 2007). Poly(1-vinylimidazole) (PVI), whose long and flexible aliphatic chains are supposed to facilitate binding to large endotoxin molecules, was introduced onto a silica gel support by controllable graft polymerization to prepare PVI-grafted-SiO_2_ particles for endotoxin adsorption (Li et al., 2011b). The obtained PVI-grafted-SiO_2_ particles significantly reduced endotoxin from 150 to 0.63 EU/ml. Additionally, we synthesized a new kind of imidazolium-based ionic liquid, 1-(12-mercaptododecyl)-3-methylimidazolium bromide (MDMIBr), to decorate the surface of the polydopamine-coated Fe_3_O_4_ magnetic particles, endowing the particles with broad-spectrum bacteria capture and endotoxin removal properties (Figure 4C) (Shi et al., 2020). Endotoxin removal capacities of the obtained MDMIBr-coated magnetic particles were 58 ± 3.3 and 24.8 ± 1.2 EU/mg in PBS and blood, respectively. These magnetic particles also exhibited good hemocompatibility and performed well in the removal of various species of clinically significant pathogens from the blood, such as *Staphylococcus aureus, Escherichia coli*, and the hard-to-treat bacteria of *Pseudomonas aeruginosa* and *Methicillin-resistant S. aureus*.

#### Amino Multi-Walled Carbon Nanotube

Amino multi-walled carbon nanotube is widely employed to adsorb pollutants in water, because it has a high surface area, porosity, and aspect ratio. Furthermore, amino multi-walled carbon nanotube (AMWCNT) also exhibits amphiphilic structures similar to those of PMB (Gusain et al., 2020). Once AMWCNTs are incorporated into the support materials, the interactions between AMWCNTs will be suppressed, and the immobilized AMWCNTs will exhibit an increased adsorption capacity (Ha et al., 2015). Accordingly, Zong et al. prepared novel polyvinyl alcohol-amino multi-walled carbon nanotube (PVA-AMWCNT) nanocomposite microspheres for endotoxin removal, in which PVA and AMWCNT were coupled *via* epoxy with amino groups (Figure 4B) (Zong et al., 2017). Compared with unmodified PVA microspheres, the endotoxin adsorption capacity of PVA-AMWCNT composite microspheres (114 EU/g) increased significantly, even slightly better than that of PVA-PMB microspheres (108 EU/g). Meanwhile, the low adsorption percentage of BSA (<3%) showed that the PVA-based microspheres had negligible non-specific adsorption in simulated serum.

Nanosized materials have proved favorable materials for endotoxin removal. They usually exhibit high adsorption capacity and efficiency resulting from their high surface areas and surface energies (Ali, 2012; Gusain et al., 2020). Like FcMBL nanobeads, traces of nano-sized materials entering or shedding into the blood is worrisome, because the biodistribution and degradation of these materials in the human body are poorly known. Nano-sized materials following their embedment into support materials as endotoxin adsorbents might be a prospective method to avoid materials entering the bloodstream and to maintain adsorption capacity. However, in the absence of experiments in the blood environment, PCL, PVI, and AMWCNT showed excellent potential for endotoxin removal. Additionally, MDMIBr also showed the versatility of simultaneous broad-spectrum bacteria and endotoxin removal.

### Low Molecular Compounds

#### Amino Acids

Although selective adsorption for endotoxin is thought to be derived from simultaneous hydrophobic and cationic properties, Wei et al. found that the endotoxin adsorption capacity of amino acid-based adsorbents increased with the rise of the isoelectric point and polarity of amino acid ligands (Wei et al., 2007b). Thus, basic amino acids such as histidine and lysine are attractive ligands for endotoxin adsorption. For instance, Zhang et al. prepared adsorbents by activation with a silane coupling agent and subsequent conjugation with histidine on porous silica gel. The obtained histidine-adsorbent (1 g) removed more than 90% of endotoxin when the volume of endotoxin solution was no more than 1,400 ml, with a corresponding K_d_ of 1,350 μg/L and a Q_m_ of 1.2 mg/g. Likewise, Fang et al. developed a novel lysine–cellulose adsorbent for endotoxin removal by immobilizing lysine covalently onto cellulose beads using epichlorohydrin as a cross-linker. Hemoperfusion using the adsorption column containing these adsorbents significantly removed endotoxin from 5.56 to 0.417 EU/ml in the blood of septic rabbits during 2-h extracorporeal blood purification sessions (Fang et al., 2004). Moreover, the lysine–cellulose adsorbent showed good results in mechanical strength, blood compatibility, and cytotoxicity, which suggested that the adsorbent had a high potential of clinical application for treatment of patients with severe sepsis.

Using a computer simulation method, Wei et al. found that serine-based agarose beads could form a cage structure with the phosphoric residues of endotoxin molecules *via* three couples of hydrogen bond (Wei et al., 2007b), which allowed them a better endotoxin removal ability compared with other amino acids. Accordingly, various biomaterials immobilized with L-serine were prepared for endotoxin adsorption in the blood (Gao et al., 2011; Huang et al., 2013; Zhao et al., 2020). For instance, Gao et al. immobilized L-serine on a polyvinylidene fluoride (PVDF) fiber to obtain the PVDF-Ser endotoxin adsorbent, which significantly reduced the levels of circulating endotoxin and inflammatory cytokines (namely interleukin-6 and tumor necrosis factor-α) in septic pigs by extracorporeal hemoperfusion, and improved respiratory function and consequent 72-h survival rate of these septic pigs (Gao et al., 2011). Similarly, Zhao et al. manufactured a novel endotoxin removal polyethersulfone (PES) electrospun fiber membrane by immobilizing L-serine onto a PES fiber membrane through a mussel-inspired method (Zhao et al., 2020). The obtained L-serine-PES membrane demonstrated an adsorption capacity of 1.28 EU/mg with the equilibrium adsorption time of about 1 h, and displayed an excellent selectively endotoxin removal efficiency of 0.85 EU/mg and anti-protein adsorption capacity in a serum system. These results suggested that L-serine-based membranes might be feasible biomaterials for sepsis treatment with significant cytokine and endotoxin removal and decreased protein adsorption.

Phenylalanine is a relatively strong hydrophobic and neutral aromatic amino acid with a pK_a_ of 5.48 that has a benzene ring as a lateral group (Dang et al., 2019). Thus, phenylalanine may be a potential ligand for the removal of lipid-soluble endotoxin and due to its hydrophobicity. As shown in Figure 4D, phenylalanine and heparin were immobilized onto polystyrene microspheres through the EDC chemistry for endotoxin adsorption in hemoperfusion. The obtained phenylalanine-adsorbent resembles oXiris® that relies on the introduction of heparin to enhance its hemocompatibility. In 5 ml of human plasma with an endotoxin concentration of 5 EU/ml, the adsorption capacity of the phenylalanine adsorbent was 15 EU/g (Dang et al., 2019).

#### Dimethylamine

**Figure 5:**
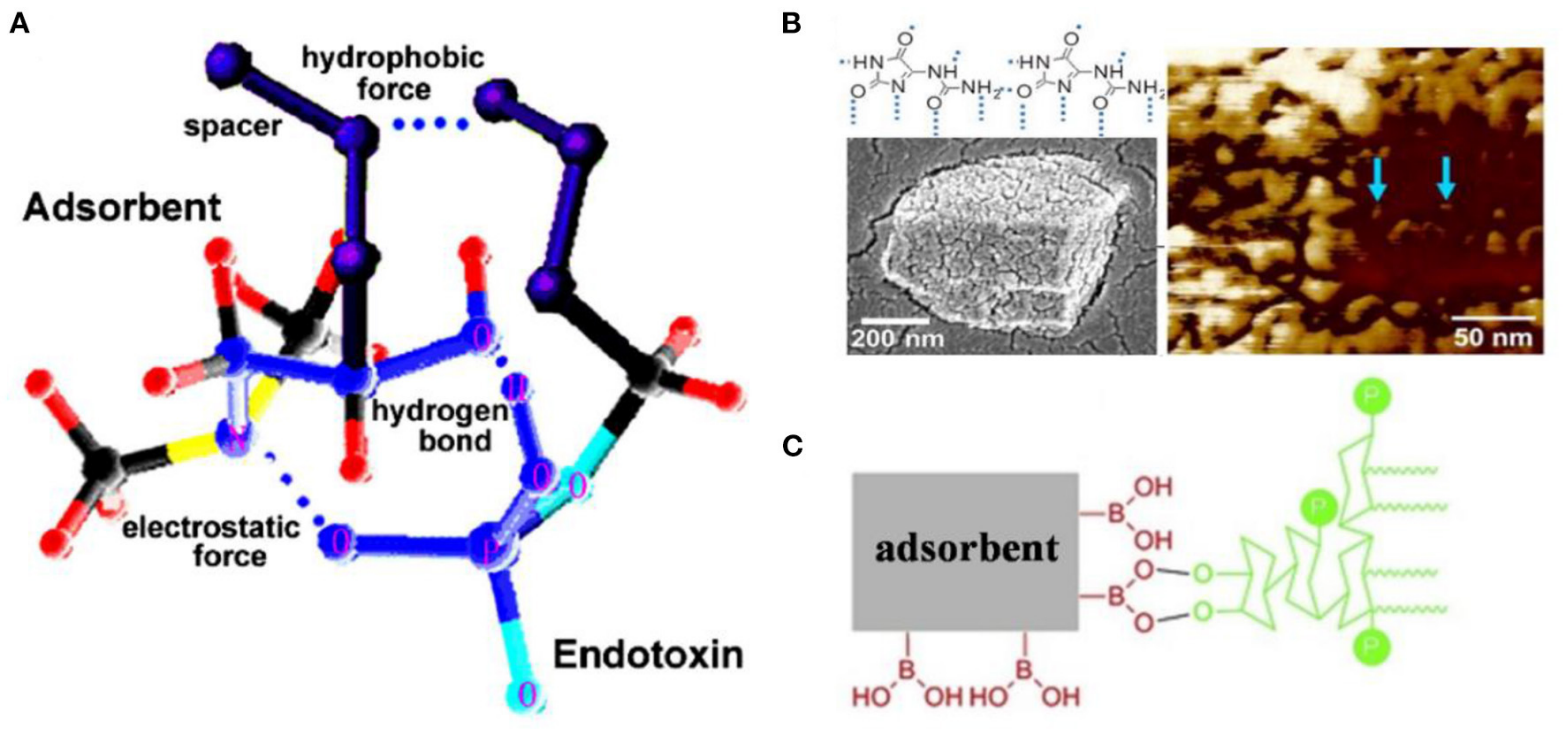
**(A)** Interaction models between endotoxin and dimethylamine with hydroxyl group at β-site. The broken lines indicate hydrogen bonds. Reproduced with permission of Elsevier (Zhi et al., 2005). **(B)** Chemical structure, scanning electron microscope, and atomic force microscopy of allantoin. Blue arrows indicate endotoxin. Reproduced with permission of American Chemical Society (Vagenende et al., 2013). **(C)** Schematic illustration for interaction between boronic acid and endotoxin. Reproduced with permission of Elsevier (Ji et al., 2019).

Zhi et al. prepared a series of endotoxin adsorbents with different dimethylamine (DMA) ligands by coating ligands on polymethyl methacrylate (Zhi et al., 2005). They found that the adsorption capacity of endotoxin increased almost eight times in the presence of a hydroxyl group at β-site of DMA ligand. In fact, the phosphate group in endotoxin could bind with the hydroxyl group at the β-site using hydrogen bonds to form an octatomic ring, as shown in Figure 5A. More recently, Tapouk et al. prepared a graphene oxide (GO)-based adsorbent (GO-ECH-DMA) using epichlorohydrin as a coupling agent and DMA as a ligand for endotoxin removal from aqueous solutions (Tapouk et al., 2019). The obtained GO-ECH-DMA adsorbent significantly removed 98% of endotoxin in solution with a Q_m_ of 121.47 EU/mg. Additionally, the GO-ECH-DMA adsorbent could be regenerated five times during their adsorption-desorption cycles with no significant loss in its adsorption capacity. Furthermore, the adsorption mechanism revealed that the excellent adsorption efficacy of GO-HCH-DMA adsorbent might be attributed to a combination of hydrogen-bonding, π-π stacking, and electrostatic interaction.

#### Allantoin

Since the discovery of endotoxin affinity to ribonucleic acid, nucleic acid analogs such as purine- and pyrimidine-based compounds and histamine have been used as endotoxin adsorption ligands. Allantoin, a purine-derived compound extensively used for biological material additives, can form a hydrogen bond with an endotoxin molecule through amide-groups (Vagenende et al., 2013). The micrographs of allantoin are shown in Figure 5B. When 300 mg/ml of allantoin crystal powder was directly added to an aqueous solution with 1,000 EU/ml of endotoxin, more than 99.9% of endotoxin was removed. The adsorption process fitted a two-site Langmuir model with high-affinity sites (H-sites) and ultrahigh-affinity sites (U-sites). Compared with H-sites (Q_m_ = 3 × 10^7^ EU/g, Ka = 1 × 10^7^ M^−1^), U-sites demonstrated a lower Q_m_ (4 × 10^5^ EU/g) but a higher K_a_ (1 × 10^10^ M^−1^) (Yan et al., 2019). It is noteworthy that the metabolic process of allantoin in the body is still of great interest, because no allantoin-based adsorbent is available at present.

#### Diaminoethane

Sakata et al. prepared aminated cellulose nanofibers (CNF) by immobilizing diaminoethane (DAE) or PEI onto chloromethyl oxirane-activated CNF (Sakata et al., 2017), and found that the DAE-CNF had higher adsorption capacity than PEI-CNF (2.5 vs. 1.9 mg/ml) with an excellent endotoxin adsorption affinity (K_d_ 2.1 × 10^−11^ mol/L). The obtained DAE-CNF reduced the concentration of contaminated LPS from 160 to a level below 0.1 EU/ml and did not affect the recovery of protein with isoelectric points from 4.9 to 11, which might be attributed to the suitable cationic properties and high surface ratio of the adsorbent.

#### Boronic Acids

Boronic acids can be used as ligands for chromatography based mainly on the interaction between boronic acids and cis-diol compounds, which is usually referred as boronate affinity chromatography (Liu, 2006). Ji et al. designed boronic acid-functionalized mesoporous silica-coated magnetic microspheres to interact with cis-diols presented in endotoxin molecule for facile endotoxin removal from plasmid DNA (Figure 5C) (Ji et al., 2019), and 5 mg of the magnetic microspheres removed almost all endotoxin from 1 ml of testing solution with an initial concentration of 0.1 EU/ml, and the adsorption capacity was calculated to be 60.84 EU/g.

#### Metallic Compounds

Various metal salt solutions can induce the precipitation of endotoxin from clarified cell lysates containing plasmid DNA with high endotoxin removal up to 80% (Ongkudon and Danquah, 2011). A crystalline adsorbent of calcium silicate hydrate was developed for endotoxin removal from aqueous solutions (Wang et al., 2005; Zhang et al., 2005). With the presence of electrolytes, the crystalline adsorbent removed more than 99% of endotoxin at low and high endotoxin concentrations. The adsorption mechanisms for crystalline calcium silicate hydrate may be attributed to Ca^2+^ cationic sites and structural properties of the adsorbent. Moreover, metallic compounds or magnetic nanoparticles could collaborate with other endotoxin adsorbents such as chitosan microspheres to enhance their endotoxin adsorption capacities significantly (Figure 3D) (Yi et al., 2012a,b; Konwar et al., 2019). For example, the amino-reserved magnetic chitosan microsphere prepared from chitosan and Fe_3_O_4_ exhibited a high endotoxin adsorption capacity of 1,792.1 EU/g with an equilibrium time of 40 min (Yi et al., 2012a).

Overall, few materials derived from low molecular compounds showed high affinity to endotoxin, which mainly depended on hydrogen bonds instead of electrostatic interactions. The notable structures between endotoxin and serine or DMA might also reinforce the combination of these compounds with endotoxin. Boronic acids demonstrated a distinct binding site with endotoxin from the others but with a lower capacity. Further studies should shed more light on the endotoxin adsorption mechanism and binding sites of these adsorbents.

## Challenges and Future Research Directions

Sepsis caused by endotoxin remains a significant healthcare challenge worldwide. In this review, we focus on current achievements in the development of endotoxin adsorbents. Table 2 shows some biomaterials that are superior in terms of adsorption affinity, capacity, or biocompatibility. Generally, both macromolecules (such as chitosan, MBL, ε-PL, PVI, and PCL) and low-molecular-weight compounds (such as allantoin, DMA, and DAE) can act as endotoxin ligands. However, several issues and challenges remain to be addressed in this area of research.

**Table 2:** Comparison of adsorbents for endotoxin.

**Ligand**	**Substrate**	**Langmuir adsorption isotherm**		**Endotoxin clearance**			**Biocompatibility**	**References**
		**Q_**m**_**	**K_**d**_**	**Endotoxin Solvent**	**Sorbent concentration**	**Initial/final endotoxin concentration**		
**Natural macromolecule**								
Chitosan	Chitosan membrane	0.28 mg/mL	4.0 × 10^−11^ mol/L	Human IgG solution	13.4 cm^2^ membrane filtrated with 12 mL solution	116.4/4.1 EU/mL	N	Machado et al., 2006
Chitosan	PVDF membrane	21,400 EU/g	0.5 EU/mL	Protein solution	0.0565 m^2^ membrane filtrated with 20 mL solution	BSA: 5.0/0.73 EU/mL Lysozyme: 5.0/1.78 EU/mL	N	Sun et al., 2005
Chitosan FeCl_3_FeCl_2_	Chitosan nanoparticle	NA	NA	Protein solution	20 mg/mL nanoparticles incubated in solution	2/ <0.02 μg/mL	N	Konwar et al., 2019
Chitosan Fe_3_O_4_	Chitosan microsphere	2210.2 EU/g	0.0213 EU/mL	NA	0.05 g microsphere incubated in 2 mL solution	17.20/9.58 EU/mL	N	Yi et al., 2012b
Chitosan Fe_3_O_4_	Chitosan microsphere	1792.1EU/g	0.448 EU/mL	NA	25 mg microspheres incubated in 2 mL solution	73.70/51.30 EU/mL	N	Yi et al., 2012a
Chitosan	Hydrogel	NA	NA	Blood	2 g hydrogels filled into a syringe with a 10 mL blood	30.0/11.0 EU/mL	Y	Li et al., 2020
LBP	PHA particle	NA	NA	Protein solution	50 μL particles incubated in 1.0 mL solution	50/0.05 EU/mL	N	Li et al., 2011a
Lysozyme	Agarose bead	NA	NA	Buffer solution	100 μL sorbent incubated in 1 mL solution	75.00/6.75 ng/mL	N	Levashov et al., 2019a
Lysozyme	Agarose bead	NA	NA	Buffer solution	100 μL sorbent incubated in 1 mL solution	50.0/5.9 ng/mL	N	Levashov et al., 2019b
P16	MSP	NA	NA	Protein solution	Nanospheres incubated in solutions at 1 mg/mL	12,500/undetected EU/mL	N	Tang et al., 2020
ε-Poly-L-lysine	Cellulose particle	NA	1.1 × 10^−11^ mol/L	Protein solution	1 mg particles incubated with 2 mL solution	32,000/ <10 pg/mL	N	Todokoro et al., 2002
**Synthetic macromolecule**								
PCL	PCL nanoparticle	NA	NA	Aqueous solution	50 μg/mL PCL nanoparticles incubated with solution	5 × 10^5^/1.06 × 10^5^ EU/mL	N	Donnell et al., 2016
PCL nano-particle	Cellulose acetate membrane	2.8 × 10^6^ EU/mL	NA	Aqueous solution	1.8 cm^2^ membrane filtered with solution	270/approximate 3 μg/mL	N	Razdan et al., 2019
PVI	Silica gel particle	3.5 × 10^6^ EU/g	NA	NA	50 mg particle incubated with 25 mL solution	30/0.07 EU/mL	N	Li et al., 2011b
MDMIBr	Fe_3_O_4_ polydopamine	58,000 EU/g	NA	Blood	NA	17.7/14.6 EU/mL	Y	Shi et al., 2020
**Synthetic macromolecule**								
Histidine	Silica gel particle	1.2 mg/g	1.35 μg/mL	Blood	1.0 g particles circulated with 1,400 mL blood	5/0.6 EU/mL	N	Zhang et al., 2007
L-serine	Agarose bead	NA	NA	Blood	0.13 g bead incubated with 0.6 mL serum	1.011/0.219 EU/mL	N	Wei et al., 2007b
L-serine	PVDF membrane	NA	NA	Blood	13 m^2^ membrane circulated with 80 mL blood	100.0/53.7 EU/mL	Y	Gao et al., 2011
L-serine	Polysulfone membrane	NA	NA	Blood	100 cm^2^ membrane incubated with 10 mL blood	0.465/0.093 EU/mL	N	Huang et al., 2013
L-serine	PES membrane	2,640 EU/g	0.332 EU/mL	NA	NA	NA	Y	Zhao et al., 2020
L-Phe	Polystyrene microsphere	25.15 EU/g	NA	NA	NA	NA	Y	Dang et al., 2019
Allantoin	–	H: 3 × 10^7^ EU/g U: 4 × 10^5^ EU/g	H: 1 × 10^−7^ mol/L U: 1 × 10^−10^ mol/L	Protein solution	300 mg/mL allantoin incubated with solution	1,000/ <1 EU/mL	N	Vagenende et al., 2013
DMA	PMMA bead	NA	NA	Blood	0.5 mL beads incubated with 4 mL blood	1.310/0.401 EU/mL	Y	Zhi et al., 2005
DMA	Agarose bead	3.30 mg/mL	1.79 × 10^−2^ mg/mL	NA	NA	NA	N	Wei et al., 2007a
DMA	Graphene oxide	121,470 EU/g	3.257 EU/mL	NA	NA	NA	N	Tapouk et al., 2019
DAE	CNF	1.1–2.5 mg/mL	2.1–8.5 × 10^−11^ mol/L	Protein solution	0.2 g adsorbent incubated with 4 mL solution	160/ <0.01 EU/mL	N	Sakata et al., 2017

*Part of data are estimated form literature*.

*NA data are not available according to the published papers. Y indicates “have been reported,” N indicates “not mentioned.”*

*PVDF, poly (vinylidene fluoride); BSA, bovine serum albumin; LBP, Human lipopolysaccharide-binding protein; PHA, polyhydroxyalkanoate; P16, a LPS specific binding peptide from Limulus anti-LPS factor; MSP, amine-functionalized mesoporous silica nanospheres; PCL, Poly-ε-caprolactone; PVI, Poly(1-vinyl imidazole); MDMIBr, 1-(12-mercaptododecyl)-3-methylimidazolium bromide; PES, polyethersulfone; Phe, phenylalanine; PMMA, polymethyl methacrylate; DMA, dimethylamine; DAE, diaminoethane; CNF, cellulose nanofiber*.

### Affinity and Capacity *in vivo*

Endotoxin can be found in both the blood circulation and extravascular tissues, but it is mainly distributed and functions in the latter. Endotoxin can move into the blood vessels from the interstitial spaces *via* various routes. This ensures that blood levels of endotoxin and inflammatory mediators after blood purification sessions frequently return to pretreatment baseline values (Carlsson et al., 2009; Romaschin et al., 2017). To reduce endotoxin in the interstitial tissues, hemoperfusion relies on rapid clearance in the blood and then equalizes the concentration between endovascular and extravascular spaces (Romaschin et al., 2017).

The adsorbents with similar or even higher adsorption capacity than PMX® were regarded as successes. However, these results should be cautiously interpreted because PMX® demonstrated poor outcomes for patients with severe sepsis (Dellinger et al., 2018). As illustrated in a post-analysis of the EUPHRATES trial, PMX® did not benefit patients with blood EAA levels >0.9 (namely, 10 ng/ml) (Romaschin et al., 2017; Dellinger et al., 2018). The total adsorption capacity for one column of PMX® was calculated to be between 10 and 20 μg, suggesting that one column can purify the blood with an initial endotoxin concentration of 4 ng/ml, assuming that the total blood volume is 5 L and that the extravascular tissue endotoxin is neglected (Romaschin et al., 2017). These results are similar to the results of previous clinical trials (Dellinger et al., 2018; Klein et al., 2018). Therefore, recommended adsorption capacity of an endotoxin adsorption column should be >50 μg to achieve better therapeutic outcomes. Notably, several adsorbents with high endotoxin adsorption capacity were found to only work at high endotoxin levels *in vitro* (Harm et al., 2014; Ankawi et al., 2018). These findings strongly suggest that further research studies are needed to verify the adsorption behaviors of these materials at lower endotoxin concentration in the blood. The K_a_ between PMB and endotoxin was calculated to range between 3 × 10^5^ M^−1^ and 2.1 × 10^6^ M^−1^ (Basauri et al., 2020). Therefore, endotoxin adsorbents with a K_d_ < 10^−7^ mol/L are recommended.

### Multifunctional Nature

Given the complicated physiopathologic processes that occur during sepsis, adsorbents such as oXiris® with multiple functions may address several therapeutic targets in severe sepsis, the most common cause of acute kidney injury in intensive care unit. These targets include endotoxin adsorption, cytokine elimination, and renal replacement treatment (RRT) (van der Slikke et al., 2021). In the previous study, crosslinked chitosan hydrogels could cleanse both Gram-negative and Gram-positive bacteria in the blood. Since bacteria burden is also associated with the mortality in sepsis (Chuang et al., 2010), simultaneous removal of endotoxin and bacteria with the extracorporeal technique provides a new insight into sepsis treatment. Multifunctional materials prepared by immobilizing endotoxin ligands such as chitosan, ε-PL, MDMIBr, serine, lysozyme, and MBL that act on cytokines or bacteria on high-flux dialysis membranes may also provide additional benefits.

### Biocompatibility

Deposition of blood clots and proteins on the surface of adsorbents can further activate both the complement and the innate-immune system to aggravate inflammation and coagulation responses during blood purification procedures, making it necessary to use anticoagulants in extracorporeal circuits to maintain their patency (Harm et al., 2019). However, critically ill septic patients often develop comorbid coagulation disorders and are, thus, at high risk of bleeding because of the administration of anticoagulants. oXiris® is hypothesized to be available for anticoagulant-free RRT, because it may prevent cells and proteins from depositing onto membrane surfaces and inhibit thrombogenesis by grated heparin layers (Kessler et al., 2013), although a recent clinical trial did not observe the benefits of heparin layers on the oXiris® membrane in prolonging the lifespan of this filter (Li et al., 2021). Alternatively, we designed anticoagulant hydrogel microspheres as an extracorporeal anticoagulant device, which could be used in hemodialysis and hemoperfusion sessions to protect the extracorporeal circuits from blood clotting without the administration of systemic heparinization. This unique device mainly performed its anticoagulant property by adsorbing the coagulation factors VIII, IX, and XI to provide transient blood thinning when placed in the extracorporeal circuit before hemofilters (Song et al., 2021). Therefore, septic patients receiving blood purification sessions using the self-anticoagulant endotoxin adsorbent cartridge or the joint application of endotoxin adsorbents with an extracorporeal anticoagulant device are theoretically at lower bleeding risk than those receiving conventional blood purification sessions by non-anticoagulant endotoxin adsorbent cartridge.

Matrices for adsorbents are also involved in adsorption selectivity and biocompatibility. However, the most commonly used matrices such as sepharose and cellulose in this field can activate the complement system powerfully because of their abundant hydroxyl groups. The pore size and surface ratio of matrices have also been proven to impact protein capture onto adsorbents (Todokoro et al., 2002; Sakata et al., 2017). Although nanoparticles (especially metallic nanoparticles) have gained immense interest as the matrices of adsorbents resulting from their excellent electrical properties, the toxicity and adverse effects of the use of metallic nanoparticles have been reported (Asghar et al., 2021). Nanoparticles are usually designed to be injected into the bloodstream directly, making it extremely important to assess the behaviors of nanoparticles with different ligands in biological fluids (Abd Ellah and Abouelmagd, 2017). In a word, it is of great significance to conduct *in vivo* animal experiments or *in vitro* biocompatibility experiments to assess the biocompatibility (such as thrombin generation, platelet activation, hemolysis, coagulation activation, and protein adsorption) of the developed endotoxin adsorbents in future studies.

### Detection and Research Design

Other than adsorption, endotoxin detection in the blood is also challenging as the poorly understood endotoxin-binding components and the anticoagulants can hamper it in plasma. The low concentrations of endotoxin in the blood are sometimes beyond the detection limits. Limulus amoebocyte lysate test is now the most sophisticated method for endotoxin detection, but its use in detecting endotoxin in blood samples has not yet been authorized (Harm et al., 2021). Indeed, cationic antimicrobial peptides and citrate anticoagulation will help endotoxin-neutralizing plasma components bind with endotoxin monomers to destabilize the activated structure of endotoxin supramolecules, leading to a diminution in detection accuracy. Consequently, the blood samples for endotoxin detection should be pre-treated with heparin rather than with sodium citrate.

Although clinicians hold that early intervention prior to inflammation reaching peak levels may be effective, it is a fact that the recognition of sepsis in early stage is difficult (Carlsson et al., 2009; Ankawi et al., 2018). Hemoperfusion has been regarded as an inconvenient and expensive therapy in patients with mild sepsis. However, *in vivo* animal experiments were performed as soon as septic models were created in most related studies currently. For the sake of reflection to real clinical settings, these hemoperfusion experiments with the developed endotoxin adsorbents are suggested to perform in animal models in advanced-stage sepsis until the reliable detection of endotoxin in blood emerges. In addition, as endotoxin is widely distributed throughout the body, special attentions should be paid to measure endotoxin levels in tissues or organs after each hemoperfusion treatment to evaluate the overall endotoxin removal efficiency of the developed adsorbents.

## Conclusion

Hemoperfusion is a feasible technique; however, current evidence does not sufficiently support the use of extracorporeal techniques in sepsis. This review provides a comprehensive overview of the development of endotoxin adsorbents and proposes the dissociation constant and the maximum adsorption capacity of the adsorbents as necessary for assessing their potential values in future blood purification sessions. Designing multifunctional, inexpensive, and easily fabricated materials with good biocompatibility may prompt the translation from laboratory to clinical use. Hopefully, as the performances of such adsorbents continue to improve, further studies are expected to overcome the problems associated with sepsis.

## Author Contributions

QY and YL designed and wrote the manuscript. All the authors contributed to the article and approved the submitted version.

## Conflict of Interest

The authors declare that the research was conducted in the absence of any commercial or financial relationships that could be construed as a potential conflict of interest.

## Publisher's Note

All claims expressed in this article are solely those of the authors and do not necessarily represent those of their affiliated organizations, or those of the publisher, the editors and the reviewers. Any product that may be evaluated in this article, or claim that may be made by its manufacturer, is not guaranteed or endorsed by the publisher.
